# Periodontal disease linked to cardiovascular disease

**DOI:** 10.4103/0975-3583.70925

**Published:** 2010

**Authors:** Rajiv Saini, Santosh Saini, Sugandha Sharma

**Affiliations:** *Departments of Periodontology and Oral Implantology, India*; 1 *Department of Microbiology, Rural Medical College, Loni, Maharashtra, India*; 2 *Department of Prosthodontics, Rural Dental College, India*

Sir,

Periodontitis is a destructive inflammatory disease of the supporting tissues of the teeth and is caused either by specific microorganisms or by a group of specific microorganisms, resulting in progressive destruction of periodontal ligament and alveolar bone with periodontal pocket formation, gingival recession, or both.[1] Periodontitis has been proposed as having an etiological or modulating role in cardiovascular and cerebrovascular disease, diabetes, respiratory disease, and adverse pregnancy outcome, and several mechanisms have been proposed to explain or support such theories and oral lesions are indicators of disease progression and oral cavity can be a window to overall health and body systems. One of these mechanisms is based on the potential effects of the inflammatory phenomenon of periodontitis on the systemic dissemination of the locally produced mediators such as C-reactive protein (CRP), interleukins-1 beta (IL-1β) and -6 (IL-6), and tumor necrosis factor-alpha (TNF-α;).[2] Periodontitis creates a burden of bacterial pathogens, antigens, endotoxins, and inflammatory cytokines that contribute to the process of atherogenesis and thromboembolic events. Of all the aspects of periodontitis, the role of bacterial pathogen is more contributing in terms of contributing factor for cardiovascular diseases. In response to infection and inflammation, certain persons may exhibit greater expression of local and systemic mediators and may thereby be at increased risk for atherosclerosis. The atherosclerosis process may result in decreased arterial patency and/or decreased compliance of the vessel. Ultimately, atherosclerotic lesions may fissure and/or rupture, resulting in occlusion of the vessel lumen, precipitating a myocardial infarction or stroke. The difficulty in drawing a cause and effect relationship between periodontitis and cardiovascular disease stems from the fact that the two groups of diseases share many risk factors. Risk factors, such as smoking, genetics, stress, and increasing age, could independently lead to periodontal disease and to cardiovascular disease in a group of patients, possibly leading to the incorrect assumption that the two diseases are linked. Three notable mediators that lead to tissue destruction and disease are IL-1, TNF-α, and MMPs. Both IL-1 and TNF-α are proinflammatory cytokines and are produced by several cell types with a diverse array of activities. Studies have demonstrated that they are involved with inflammation and connective tissue breakdown and can limit repair of periodontium and myocardium. Specifically, their role in inflammation includes the stimulation of adhesion molecule and chemokine expression, and production of other inflammatory mediators such as prostaglandin E2. The chronicity of periodontal disease provides a rich source of subgingival microbial and host response products and effects over a long-time period. Two main processes may provide a causative link between these two diseases: the lipopolysaccharide and monocyte-related responses.[3] Thus, it can be concluded that relationship between oral disease (specifically periodontitis) and atherosclerosis/coronary heart disease exists and indicates that periodontitis plays a considerable role in the pathogenesis of atheroma formation, as well as in cardiovascular events.
